# Breast Cancer and Women Veterans: What Is the Impact of Mental Health on Screening Rates?

**DOI:** 10.3390/medicines10010001

**Published:** 2022-12-20

**Authors:** Saranya Prathibha, Anders D. Westanmo, Jane Yuet Ching Hui, Katie Westanmo, Amy A. Gravely, Todd M. Tuttle, Christopher J. LaRocca

**Affiliations:** 1 Minneapolis Veterans Affairs Health Care System, One Veterans Drive, Minneapolis, MN 55417, USA; 2 Department of Surgery, University of Minnesota, 420 Delaware St SE, Mayo Mail Code 195, Minneapolis, MN 55455, USA

**Keywords:** breast, screening, veterans, mental health

## Abstract

**Background:** The proportion of women Veterans are increasing and, as such, access to high-quality breast cancer care is important. Prior studies have shown that rural location, age, and a mental health diagnosis negatively impact breast cancer screening rates. **Methods:** We aimed to retrospectively assess the impact of these risk factors on breast cancer screening adherence rates among Veterans at our institution. Women who were eligible for breast cancer screening per the United States Preventative Services Taskforce guidelines were included. **Results:** Of 2321 women, overall adherence was 78.2%. There were no significant differences in screening rates between races, various age groups, geographical distribution, and having anxiety or post-traumatic stress disorder (PTSD). However, Veterans with a diagnosis of depression were more likely to adhere to screening guidelines. Having multiple mental health diagnoses was also not a negative risk factor. **Conclusions:** Our Veteran population’s adherence rates are higher than the national average and rural location, race, age, and certain mental health disorders did not negatively affect adherence to screening mammography. Though more research is needed, screening reminders from our women’s health coordinator may have improved adherence rates and lowered disparities.

## 1. Introduction

Breast cancer remains a leading cause of mortality worldwide and its complex nature necessitates nuanced treatment decision-making based on histological subtypes, receptor status, genetic mutations, and the extent of disease [1,2]. Importantly, breast cancer screening with mammography plays a vital role in reducing breast cancer mortality [3]. However, some possible barriers to the successful completion of screening mammograms include fear of study results, lack of knowledge, long wait times, geographical access, and financial issues [4]. The Veteran population is important to study given their unique environmental and occupational exposures, and to further elucidate factors contributing to their worse physical and mental health status when compared to the civilian population [5]. In particular, women make up an increasing proportion of the Veteran population and, as such, continued efforts are needed to ensure access to high-quality breast cancer care. The Veterans Affairs (VA) healthcare system consists of a network of tertiary care centers linked with subsidiary community-based outreach centers (CBOC). Prior studies assessing both civilian populations and Veterans have shown that age, rurality, and a mental health diagnosis have a negative impact on breast cancer mammographic screening rates [6,7,8,9,10]. We aimed to assess the impact of these risk factors on screening adherence rates among Veterans at our institution. 

## 2. Materials and Methods

A retrospective study was completed to assess breast cancer screening among women Veterans at a single metropolitan tertiary care center and its associated CBOCs. Women who were eligible for breast cancer screening per the United States Preventative Services Taskforce guidelines (biennial screening mammogram for women ages 50–74 as of October 2021) were included. Adherence rates were defined as obtaining a screening mammogram during the study period of October 2019–October 2021. If an eligible Veteran did not receive a mammogram within the study period, they were considered non-adherent. The impact of geographical distribution, race, age, and mental health disorders (including post-traumatic stress disorder (PTSD), depression, and anxiety) on screening rates were evaluated. Administrative data were utilized to identify patients with active mental health diagnoses during the study period. Patients with a history of breast cancer or bilateral mastectomies were excluded. A univariate analysis was completed using a Pearson chi-square test. This work has been approved by the appropriate ethical committee at our institution and due to the retrospective nature of the data of this quality-improvement project, informed consent was waived. 

## 3. Results

Through retrospective chart review, we identified a total of 2321 women Veterans who were eligible for breast cancer screening between October 2019 and October 2021. Our cohort had a median age of 61 years, was 57% urban, and had a racial distribution of 89% White, 8% Black, 1% Asian, 2% American Indian/Alaska Native, and 1% Native Hawaiian or Other Pacific Islander. Baseline rates of PTSD, anxiety, and depression were 18%, 31%, and 41%, respectively. Overall adherence was 78.2%. Urban women had similar rates of adherence to rural and highly rural women (77%, 79%, and 80%, *p* = 0.54). There were no significant differences in screening adherence rates between racial groups (*p* = 0.22) or among all eligible age ranges (*p* = 0.11). Diagnoses of PTSD or anxiety were not associated with worse adherence rates when compared to patients without these diagnoses (PTSD 77% and non-PTSD 79%, *p* = 0.37; anxiety 79% and non-anxiety 78%, *p* = 0.51). Veterans with a diagnosis of depression were significantly more likely to adhere to screening guidelines, with adherence rates of 81% vs. 76% (*p* = 0.01). Women Veterans with more than one mental health diagnosis (within the categories listed above) did not have any difference in adherence rates compared to women with one mental health diagnosis (*p* = 0.45; Table 1).

## 4. Discussion

Our Veteran population’s adherence rate to mammographic screening guidelines (78.2%) is higher than the national average (73% of the general population in 2018) [11]. Our data demonstrates that rural location, race, age, and certain mental health disorders do not negatively affect adherence to screening mammography, which is contrary to other published reports in civilian centers [6,8,9]. While the screening rates demonstrate an increase from urban to highly rural populations, we cannot draw any conclusions about causation given the retrospective nature of the study and the small sample size for certain patient groups. Interestingly, we found that a diagnosis of depression was associated with a significantly higher adherence rate, possibly related to utilization of healthcare resources. A study by Lairson et al. suggested that the military may contribute to racial equality among breast cancer screening, which our findings also support as we observed no disparity in screening rates across racial groups [7]. The screening rate in the Asian population in this study was low but the small sample size of that group was limiting and further study will be needed on this point. Our institution utilizes a women’s health coordinator with designated liaisons at each CBOC, who send reminders approximately each month of provider-specific patient lists with mammogram due dates. This program may be contributing to our high adherence rate; however, further studies throughout the VA system are needed. Limiting factors of this study include the inability to obtain retrospective data prior to 2013 when the role of the women’s health coordinator was more limited, difficulty in accounting for Veterans who obtain medical care outside of the VA healthcare system, and potential inaccuracies of using administrative data to obtain active mental health diagnoses. Furthermore, our data from this metropolitan area may not be representative of the entire VA system.

## 5. Conclusions

Further research is still needed, but the role of the women’s health coordinator to facilitate provider-specific active reminders for breast cancer screening has likely helped to decrease any disparities across age/race/location and may even serve to increase adherence rates in patients with mental health diagnoses who are historically at risk for having lower cancer screening rates. This model warrants further study and implementation at other VA centers.

## Figures and Tables

**Table 1:** Screening rates of women Veterans based on rurality, race, age, and mental health disorders.

Population Characteristics	*n*	Adherence (%)	*p*-Value
**Rurality**	2320		
Urban	1312	77	0.54
Rural	953	79	
Highly rural	55	80	
**Race**	2132		
White	1893	78	0.22
Black	159	82	
Asian	14	57	
American Indian or Alaska Native	51	75	
Native Hawaiian or Other Pacific Islander	15	80	
**Age, years**	2321		
50–59	1039	77	0.11
60–69	1041	80	
≥70	241	74	
**Mental health disorder**	2321		
PTSD	427	77	0.37
Non-PTSD	1894	79	
Anxiety	711	79	0.51
Non-anxiety	1610	78	
Depression	958	81	0.01
Non-depression	1363	76	
One mental health disorder	445	78	0.45
Two or more mental health disorders	542	80	

## Data Availability

We are unable to provide data to post/submit as the information is confidential to our institution.
